# Histological verification of atherosclerosis due to bends and bifurcations in carotid arteries predicted by hemodynamic model

**DOI:** 10.1590/1677-5449.004118

**Published:** 2018

**Authors:** Rajani Singh, Richard Shane Tubbs

**Affiliations:** 1 All India Institute of Medical Sciences (AIIMS), Department of Anatomy, Rishikesh, Uttrakhand, India.; 2 Seattle Science Foundation, Seattle, WA, USA.

**Keywords:** tortuosity, bifurcations, microanatomy, hemodynamic model, tortuosidade, bifurcações, microanatomia, modelo hemodinâmico

## Abstract

**Background:**

Tortuosity and bifurcations in carotid arteries alter the blood flow, causing atherosclerosis.

**Objectives:**

The aim of the present study is to analyze the effect of variant vascular anatomy in the cervical region on development of atherosclerosis by microanatomical examination.

**Methods:**

The effect of blood flow at anomalous bends and bifurcations was observed in right carotid arteries of a seventy year old female cadaver. Fifteen histological slides were prepared from the carotid arteries and interpreted to verify predictions of atherosclerosis.

**Results:**

The model predicts atherosclerosis at bends, bifurcations and large aperture arteries. Microanatomical examination revealed presence of atherosclerosis of varying thickness at the bends and bifurcation in the right carotid arteries, as predicted. Atherosclerosis was also detected in the straight part of the wider common carotid artery. No atherosclerosis was observed in the contralateral carotid arteries. The variant carotid vascular anatomy consisting of bends, bifurcations and wider arteries revealed that the shear stress and velocity of blood flow are reduced at these anomalous sites.

**Conclusions:**

Anatomical anomalies such as bends and branching in the carotid arteries alter the irrigation pattern and generate biomechanical forces that cause turbulent flow and reduce shear stress/blood flow velocity. Decreased shear stress and velocity causes development of atherosclerosis. Histological slides established the presence of atherosclerosis at bends and bifurcations and in wider arteries.

## Resumo

**Contexto:**

Tortuosidade e bifurcações das artérias carótidas alteram o fluxo sanguíneo, causando aterosclerose.

**Objetivos:**

O objetivo do presente estudo foi analisar o efeito de anatomia vascular variante na região cervical sobre o desenvolvimento de aterosclerose via exame microanatômico.

**Métodos:**

O efeito do fluxo sanguíneo em dobras e bifurcações anômalas foi observado nas artérias carótidas do lado direito em um cadáver do sexo feminino de 70 anos de idade. Quinze lâminas histológicas foram preparadas a partir das artérias carótidas e interpretadas para confirmar as previsões de aterosclerose.

**Resultados:**

O modelo prevê aterosclerose em dobras, bifurcações e artérias de grande calibre. O exame microanatômico revelou a presença de aterosclerose de densidades variáveis nas dobras e bifurcação das artérias carótidas do lado direito, conforme previsto. Aterosclerose também foi detectada na parte reta da artéria carótida comum mais larga. Não foi observada aterosclerose nas artérias carótidas contralaterais. A anatomia vascular carotídea variante consistindo de dobras, bifurcações e artérias mais largas revelou que a tensão de cisalhamento (*shear stress*) e a velocidade do fluxo sanguíneo são reduzidos nesses pontos anômalos.

**Conclusões:**

Anomalias anatômicas tais como dobras e ramificações das artérias carótidas alteram o padrão de irrigação e geram forças biomecânicas que causam fluxo turbulento e reduzem a tensão de cisalhamento e a velocidade do fluxo. Tensão e velocidade menores causam o desenvolvimento de aterosclerose. As lâminas histológicas estabeleceram a presença de aterosclerose nas dobras e bifurcações nas artérias mais largas.

## INTRODUCTION

 The regulated, uniform, continuous and streamlined blood flow carried through regular-shaped carotid arteries perfuses the structures of the head and neck. However, anatomical anomalies such as siphons/bends and branching in the common carotid/external carotid/internal carotid arteries (CCA/ECA/ICA) alter the irrigation pattern and generate biomechanical forces that cause turbulent flow and reduce shear stress/blood flow velocity. 1 The reduction in shear stress and blood flow velocity causes endothelial injury. Injury to the endothelium causes endothelial dysfunction, modifying the internal vascular structure by activating accumulation of atherosclerosis leading to many fatal clinical consequences. Many factors contribute to atherosclerosis including high blood pressure, tobacco smoke, diabetes, and high levels of cholesterol 2 in the blood. Turbulent blood flow at bends and bifurcations disturbs the balance between vasodilatation and vasoconstriction, inhibition and stimulation of smooth muscle cell proliferation and migration, thrombogenesis and fibrinolysis, 3 ^,^ 4 and initiates a number of processes that promote or exacerbate atherosclerosis, including increased endothelial permeability, platelet aggregation, leukocyte adhesion, and generation of cytokines. 5

 This dysfunction, characterized by impaired nitric oxide availability, is considered one of the main promoters of athero-thrombosis. 6 Endothelial dysfunction is an early marker for atherosclerosis preceding angiographic or ultrasonic evidence of atherosclerotic plaque formation. 4 Shear stress increases the expression of endothelial nitric oxide synthase signaling. Asymmetric dimethylarginine (ADMA) inhibits nitric oxide production, and elevated levels of ADMA have been associated with endothelial dysfunction and atherosclerosis. 7

 The observational studies from previous research work reveal that tortuosity comprising bends and bifurcations/branching points causes the velocity/stress of blood flow to reduce, causing endothelial damage, which in turn results in endothelial dysfunction at these anatomically anomalous structures. This dysfunction is a major factor in exacerbation of atherosclerosis, as cited above. 

 Therefore, the aim of this study is to verify whether abnormal anatomy containing cervical siphons bends/tortuosity and bifurcations in the CCA, ECA and ICA and changing circulatory dynamics causes atherosclerosis. This is examined experimentally by histological study. This study will be of paramount importance for clinicians, vascular surgeons and anatomy. 

## MATERIAL AND METHODS

 Severe tortuosity of the ECA and ICA was detected in a seventy-year-old female cadaver, one of a total of 14 cadavers examined. Initially, theory was applied to attempt to comprehend the influence that anomalous blood flow patterns through the siphons/bends and bifurcations in the tortuous carotid arteries would have on normal biomechanical forces. The inferences of this theory were correlated with development of atherosclerosis. This concept was then experimentally verified against histological slides and physically by hand lens. 

 The drag force and velocity of the blood flow through cross sections of bends/ siphons or bifurcations/branching points are altered because of directional variations and momentary obstructions. Therefore, if the force exerted by the pumping heart is F_d_, the drag stress S_d_ and area of cross section of the artery A, then Sd = F_d_/A. (Here drag stress is large for smaller area and vice-versa for a constant force F_d_ due to pumping of the heart.) 

 The Sd in a viscous flow can be derived from Stoke’s theorem depending on direct proportion to the velocity at the center (V_c_) and inversely as radius of the vessel (Ra), as - S_d_ ∝ V_c_)/R_a_. 

 This means Sd will be higher for higher Vc and lower for higher Ra. This is for a straight path along an artery. 

 However, in the presence of bends and bifurcations, the theory stated above must be modified as follows: The velocity and stress due to blood flow will be reduced at bends and bifurcations due to directional variation and obstructive flow. The variation (reduction) in velocity is directly dependent on the angle of deviation of flow direction. This means that if the angle of deviation of flow is 0, the variation (reduction) in velocity will be 0. If variation in the angle is 30º, the reduction in velocity will be 13.4% (0.134) and for an angle of deviation of 60º, the reduction will be 50% (0.5). However, as illustrated above, Sd is low for low velocities and vice-versa. So, for lower deviation in angle, Sd too will be low. However, the arteries (ECA/ICA) follow an S-shaped pattern, consisting of multi-bend structures, so the flow will suffer from a cascaded effect of directional change and obstructive flow, resulting in substantial cumulative fall in velocity/stress depending on number of bends. Therefore, the velocity of blood flow and the drag stress due to bends and branching points and/or wider arteries decrease progressively. Notwithstanding, the variability of Sd is different from the variability of velocity because in addition to being dependent on velocity, Sd is also dependent on area so on artery radius. Therefore, even if there is appreciable reduction in Sd due to deviation, but the radius is decreased, then Sd will increase, compensating the reduction. Thus, the theoretical hemodynamic model evolves as follows: 

 The blood circulation at bends, bifurcations and large aperture arteries suffers from loss of velocity, drag shear stress (under the constraint of dual dependence as explained above), and kinetic energy, causing injury to the innermost layer of the arterial wall in direct contact with the blood, the endothelium of the tunica intima, resulting in endothelial dysfunction, a major cause of atherosclerosis. 

 To assess the effects of these anatomical anomalies of bends and bifurcations on atherosclerosis, the angles, location and radii of the bends were measured as described in Table 1 . 

**Table 1:** Measurements of radius, bend angle and thickness of atherosclerosis at bends, bifurcations and straight part of CCA, ECA, ICA arteries.

**Section**	**Bend/Bi**	**Rad. (mm)**	**θ**	**T(mm)**	**Remarks**
CCA-1	St.	8.5	0	19-39	Wider
CCA-2	St.	8.5	0	50-79	Wider
CCA-3	Bi/ECA/ICA	10		7-15	Bif. ECA/ICA
ECA-1	A/Bi/STA	5.0	20	1.0-2.5	Bend/Bif.
ECA-2	Bi/LA	4.5		2-5	Wider
ECA-3	C	4.0	52	1.5-8	Bend
ECA-4	Bi/OA	2.5		1-3	Narrower
ECA-5	D	2.0	60	2-11	Bend
ECA-6	St	2.0		NIL	Straight
ECA-7	E	2.0	30	2-10	Bend
ECA-8	Bi/MA	1.5		2-8	Bif.
ECA-9	Bi of MA	1.5		1.5-2.5	Bif.
ICA-1	Aʹ	5.5	28	2-7	Bend
ICA-2	B´	5.0	48	2-15	Bend
ICA-3	St	5.0		22	Straight

CCA-1, First slide sample from common carotid artery at its origin; ECA-1, First slide sample from external carotid artery; ICA-1, First slide sample from internal carotid artery; Bend/Bi, Bends/Bifurcations; St, Straight part; A/Bi/STA, Bend name/Bifurcation/superior thyroid artery; LA, Lingual artery; C, name of the bend; Rad.(mm), Radius in millimeter; θ, angle of bend; T(mm), Thickness in mm.

 Finally, to verify the presence of atherosclerosis at bends and bifurcations in the ECA and ICA and in a large aperture artery, the CCA was analyzed by preparing histological slides and by physical observation. Six sections were cut at bends, five at bifurcations in the CCA, ECA and ICA, and four from the straight parts of these arteries. 

### Description of samples of sections

 Fifteen histological slides comprising three from the CCA, nine from the ECA and three from the ICA were processed and prepared.

1. Three sections from the CCA (CCA 1-CCA 3): First at the origin, second between the origin and bifurcation, and third at the bifurcation of the CCA into ECA and ICA.
2. Nine sections from the ECA (ECA 1-ECA 9) at bends and bifurcations: ECA-1 at first bend and branching point of the superior thyroid artery, ECA-2 at the separation of the lingual artery, ECA-3 at the third bend, ECA-4 at the origin of the occipital artery, ECA-5 at the fourth bend, ECA-6 in the straight part between the fourth and fifth bends, ECA-7 at the fifth bend, ECA-8 at the origin of the maxillary artery, and ECA-9 at the bifurcation of the maxillary artery.
3. Three sections from the ICA at bends (ICA 1-ICA 3): ICA-1 at the first bend, ICA-2 at the second bend and ICA-3 from the straight part of the ICA after the second bend.

Five micron sections were cut and stained with hematoxylin and eosin. These slides were prepared and photographed using a 5 megapixel camera attached to a PZRM-26 model microscope. “Future Winjoe” software was used for interpretation and detection of atherosclerosis. 

## RESULTS

Two inferences follow from the hemodynamic theory:

1. The larger the area, A (A = π(Ra), 2 Ra = radius) of the artery, the smaller the shear stress and velocity and vice versa.
2. The velocity of blood flow and the shear stress fall at bends and branching points. In multi-bend arteries the reduction is cumulative.

In the present case, the ECA courses through five bends (A, B, C, D, and E) at 20˚, 51˚, 52˚, 60˚, and 30˚, and the ICA travels through two bends (Aʹ, Bʹ) forming angles of 28˚ and 48˚. Therefore, owing to the directional change of the vectors **V** and **S_d_**, the velocity and stress are cumulatively reduced to 16% and 59% of their values at the start of the first bend in the ECA and ICA, respectively. 

###  Results of physical examination and microanatomical analysis of slides 

 The radii of the CCA varied from 8.5 to 10.0 mm between the origin and bifurcation into the ECA and ICA. The radii of the ECA at bends, bifurcations, and straight parts ranged from 5.0 mm to 1.5 mm between the origin and the point of termination. The ICA did not give off any branches, so its radius remained almost constant at 5.0 mm from the origin to its entry into the base of the skull. The cross-sectional area of the ECA continuously decreased from 79 mm^2^ to 7 mm^2^ due to branching as it advanced from origin to termination. Table 1 lists the variations in thickness of atherosclerosis at bends, bifurcations, and straight parts of the CCA, ECA, and ICA, in millimeters at 4X/10X magnification and 50% zoom. 

**Figure 1:**
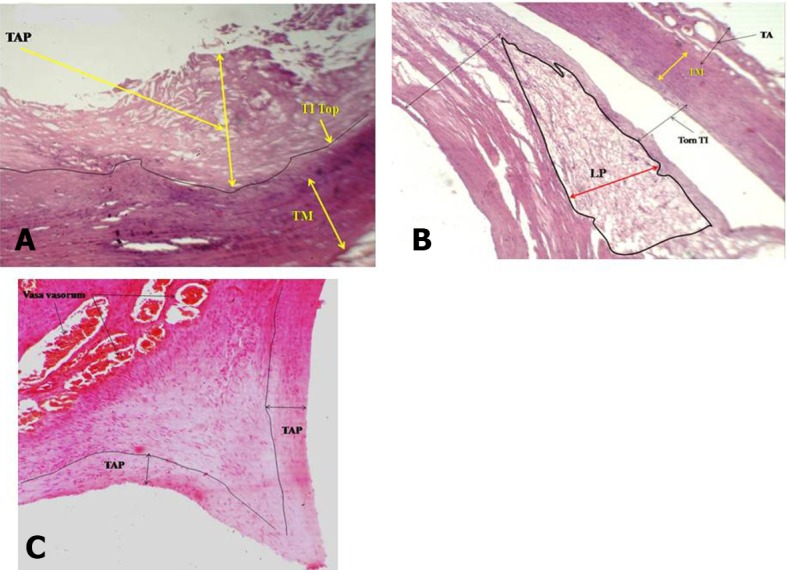
**A)** Atherosclerosis in section from origin of common carotid artery; **B)** Atherosclerosis in section of common carotid artery, from midway between the origin and bifurcation; **C**) Atherosclerosis in section from common carotid artery at bifurcation into external and internal carotid arteries; 4X magnification; TAP, Thickness of atherosclerotic plaque TM, tunica media; TI Top, tunica intima top ; Torn TI, Torn tunica intima; LP, Lipid; TI, tunica intima.

**Figure 2:**
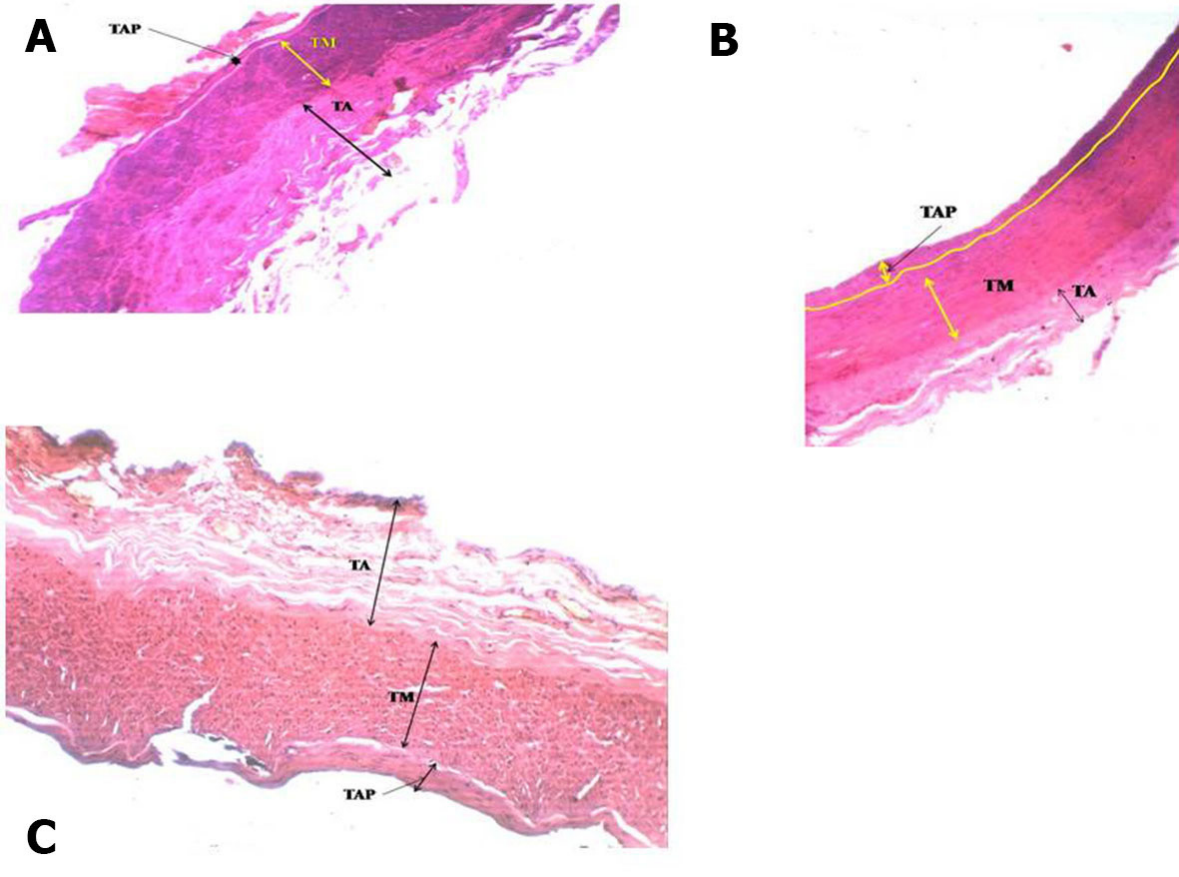
**A)** Atherosclerosis in external carotid artery at first bend and origin of superior thyroid artery; **B)** Atherosclerosis in external carotid artery at bifurcation at origin of lingual artery; **C)** Atherosclerosis in external carotid artery at 3rd bend; TAP, Thickness of atherosclerotic plaque; TM, tunica media; tunica intima; TM, tunica media; TI, tunica intima, 4X magnification.

**Figure 3:**
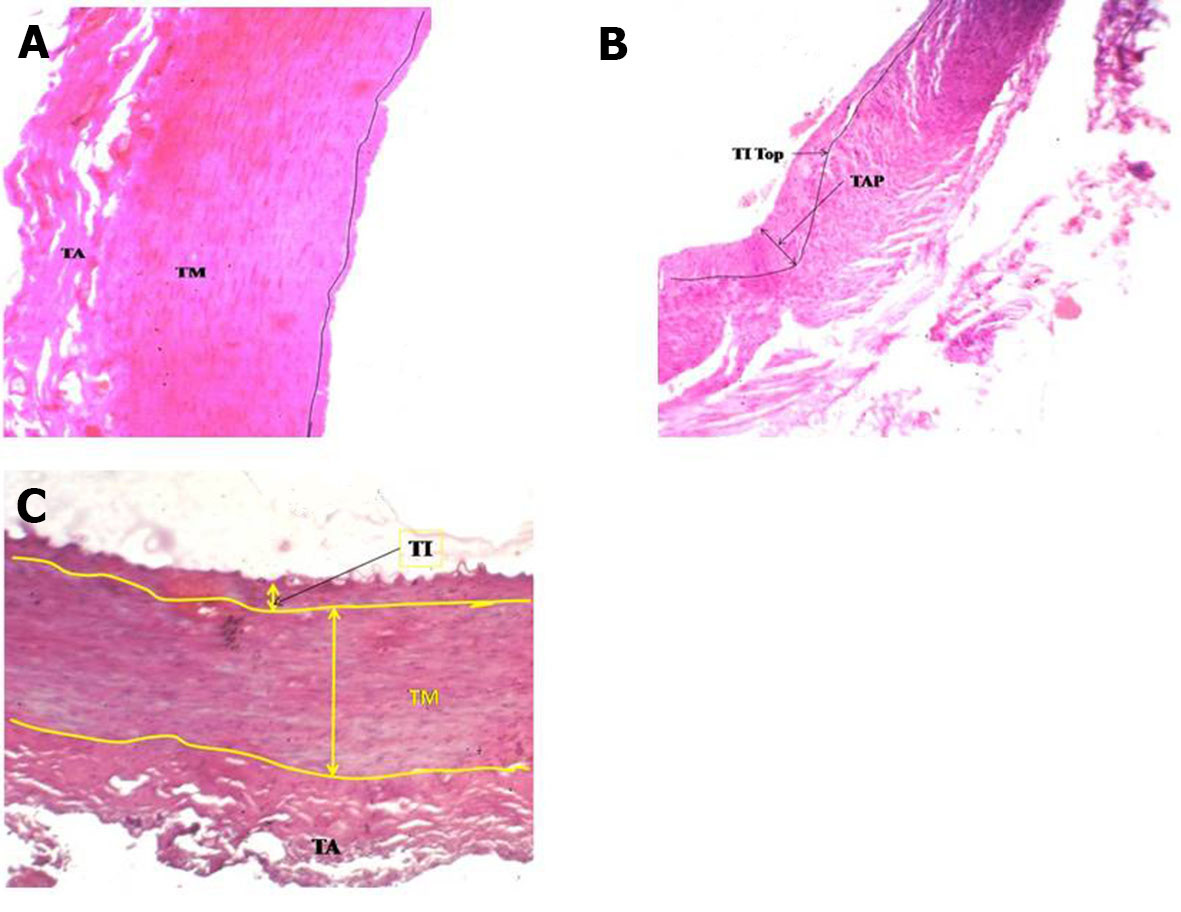
**A)** Atherosclerosis in external carotid artery at bifurcation at origin of occipital artery; **B)** Atherosclerosis in external carotid artery at 4th bend; **C)** No atherosclerosis in external carotid artery in straight part between 4th and 5th bends; TAP, Thickness of atherosclerotic plaque (10-30 mm); TM, tunica media; TA, tunica adventitia; 4X magnification.

**Figure 4:**
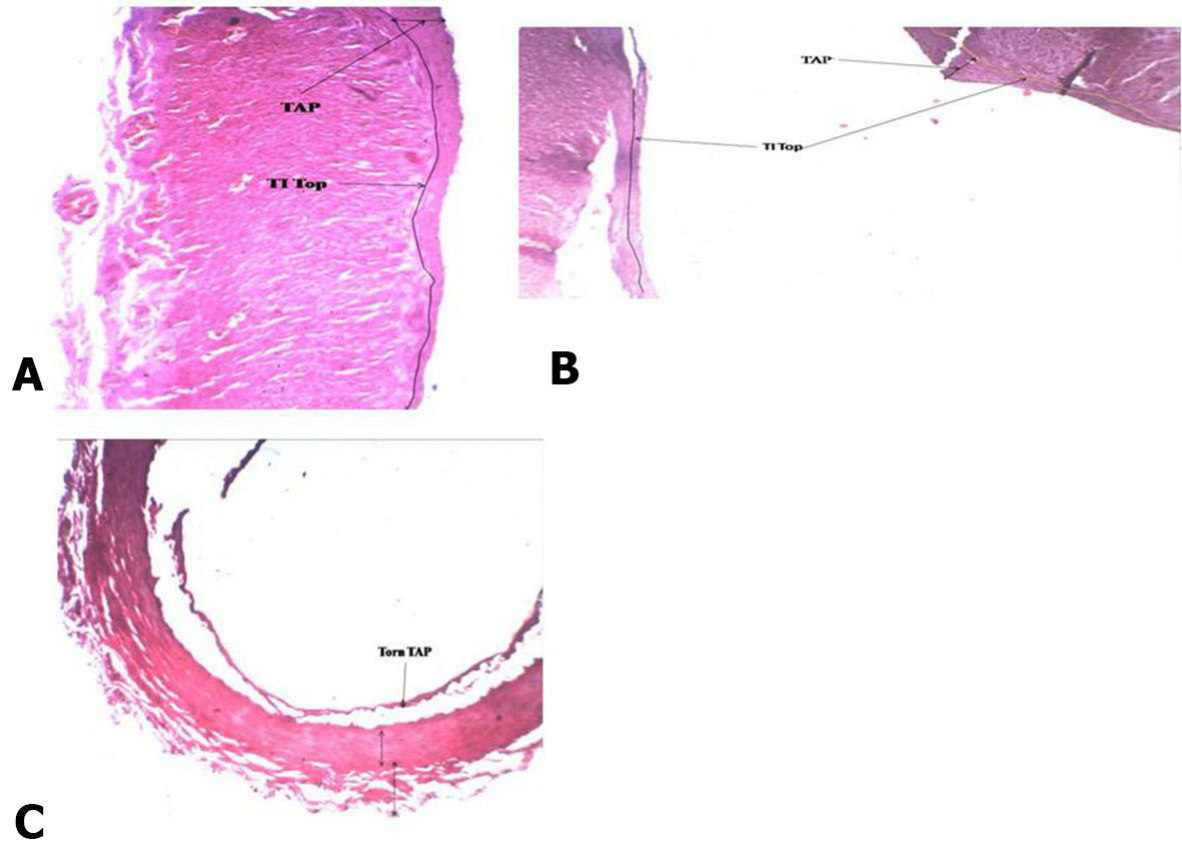
**A)** Atherosclerosis in external carotid artery at 5th bend; **B)** Atherosclerosis in external carotid artery at bifurcation of maxillary artery; **C)** Atherosclerosis in external carotid artery at bifurcation of maxillary artery TAP, Thickness of atherosclerotic plaque (2 mm-10 mm); TI Top, tunica intima top; 4X magnification.

**Figure 5:**
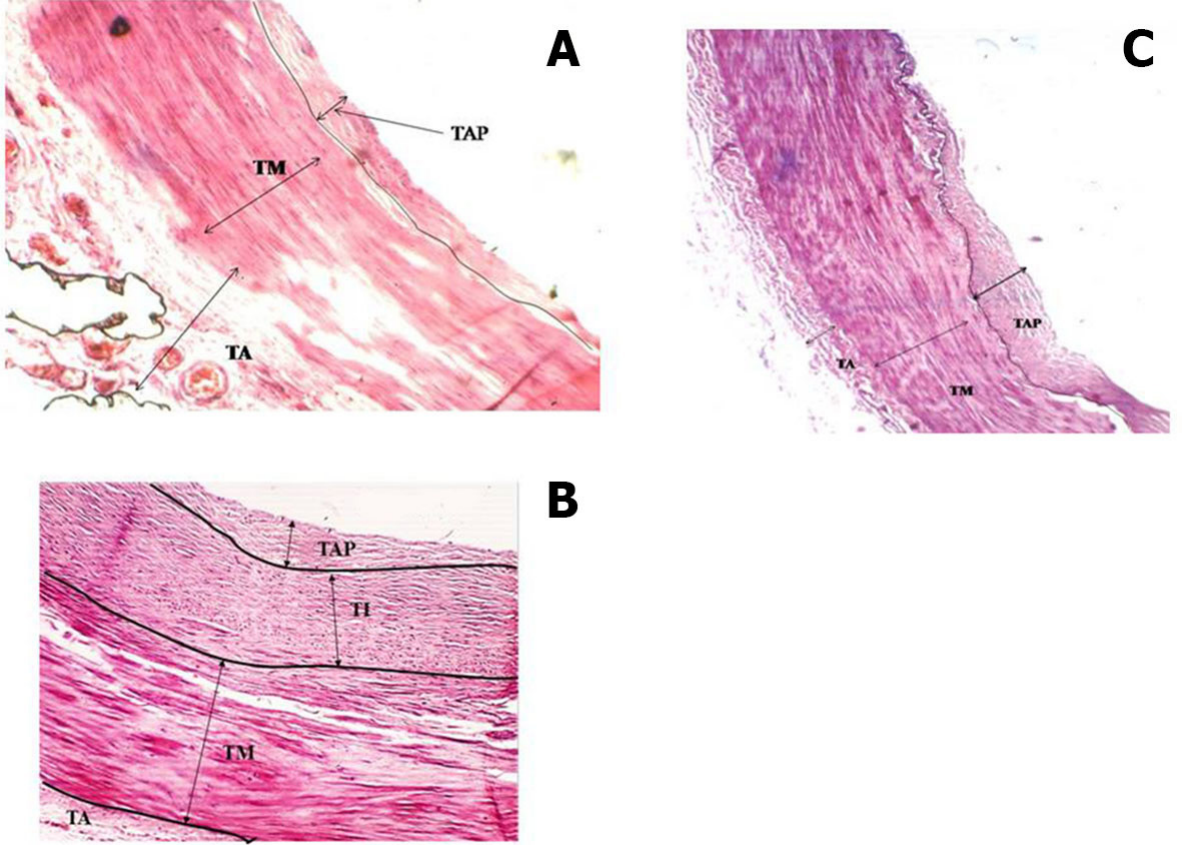
**A)** Atherosclerosis in internal carotid artery before 1st bend; **B)** Atherosclerosis in internal carotid artery at 2nd bend; **C)** Atherosclerosis in internal carotid artery beyond 2nd bend; TAP, Thickness of atherosclerotic plaque; TM, Tunica media; TA, Tunica adventitia; 4X magnification.

 Quite thick atherosclerosis ranging from 7 to 79 mm was detected in the **widest** artery, the CCA, in the straight part and at the point of bifurcation ( Figure 1 ). There were accumulations of atherogenic material ranging from 1 to 11mm thick in the ECA at bends A, C, D, E ( Figure 2 , 3 and 4 ) and at branching points ( Figure 2 , 3 and 4 ). The thickness of atherosclerotic plaque in the ICA ranged from 2 to 22 mm ( Figure 5 ). These depositional patterns possessed variable thickness of atherogenic material in the periphery of the vessel. The thickness of atherosclerotic material at bends in the ICA was greater than in the ECA. Vasa vasorum were also seen at the bifurcation of the CCA into the ECA and ICA. Physical observations and interpretation of slides were qualitatively consistent with each other. There was almost no atherosclerosis at the end in narrower arteries. There was a large amount of atherosclerosis at the bends and bifurcations and in the straight parts of the wider arteries. 

## DISCUSSION

 Kinking in ICA/ECA may be congenital or acquired lesions and are most commonly seen in elderly patients suffering from arteriosclerosis and hypertension. 8 Beigelman et al. showed that kinking/coiling of the carotid arteries can arise during embryological development and cause changes to flow dynamics. 9 The authors of the present study are of the view that tortuosity is congenital and in turn causes altered flow dynamic leading to mural changes in vessels. 

 The presence of anatomical variants such as siphons/bends, branching in the ECA/ICA and wider CCA alter the circulatory dynamics of blood flow, causing velocity and drag stress to reduce, as explained in the materials and methods section. The diminution in velocity/shear stress occurs under two conditions: firstly due to directional and obstructed blood flow at bends and bifurcations; secondly in arteries with large aperture. So the drop in velocity/stress will be seen not only at bends and bifurcations, but also in large and medium-sized arteries. Therefore, these are sites of low velocity and low shear stress in this arterial tree. The diminution of velocity/stress at bends and bifurcations have also been reported by other authors. 1 ^,^ 10 ^-^ 12 We now analyze the fall in drag stress and velocity due to the cervical siphon and branching of the ECA and ICA. The velocity and stress diminish by a factor of cosө at a single bend forming an angle ө with central axis of flow due to directional change and resolution of vectors. But the present case exhibits multi-bend, S-shaped tortuous ECA/ICA and therefore there will be cascading effect on reduction of blood flow velocity and dragging stress. This analysis proves the reduction of velocity and stress at bends/bifurcations. 

 The drag force exerted by flow on the endothelium of vessel wall, called shear stress, is critical for maintaining its function and varies with time, magnitude, and direction, according to vascular pulsatility and anatomic anomalies. Relatively straight, branchless arteries are exposed to uniform, unidirectional flow and experience relatively high shear stress (time-averaged mean value approximately 150 µN/cm^2^; equivalent to 15 dynes/cm^2^ ). In contrast, at vessel bifurcations and bends, the flow is disturbed, changes direction and pulsatility during the cardiac cycle, resulting in relatively low and/or oscillatory shear stress (time-averaged mean < 50 µN/cm^2^; equivalent to < 5 dynes/cm ^2^). 10 This supports our hemodynamic theory. However, the fall in velocity/shear stress associated with oscillatory turbulent flow due to pulsatility causes injury to endothelial layer, leading to endothelial dysfunction. According to Malek et al., 10 the endothelium is in direct contact with flowing blood and is therefore constantly exposed to the biomechanical forces that the blood exerts. In straight laminar blood flow, in general in narrow but straight and branchless arteries in particular, high velocity and high stress on the walls of the vessel prevent the atherosclerotic process and protect against endothelial dysfunction. This injury will be further accentuated by lower velocity causing more time of contact exerting lower dragging stress on the endothelium layer. At bifurcations, the flow volume per second is divided into two parts in the ratio of the areas of cross section of the two arteries. But the stress or pressure is dependent on the area of cross section of the artery; therefore the stress/velocity would be higher in narrower arteries than wider ones. However, the main artery from which the branch arises is normally wider so the stress drops, causing atherosclerosis. As revealed by several studies, 1 ^,^ 13 potentially preferable sites for atherosclerosis are at bifurcations and bends/tortuous courses that present lower shear stress and lower flow velocity under turbulent conditions of pulsatile blood flow in the ECA and ICA. However, atherosclerosis was also observed in arteries with straight courses and large apertures (CCA) ( Figure 1 1B) in the present study. Simultaneously, atherosclerosis remained absent from distal arteries with small aperture, even when there were bends/tortuous courses or bifurcations. This is because pressure from the pumping of the heart is approximately constant throughout the body, so the blood flow velocity and drag stress will be high in narrower arteries even at bends and bifurcations, in contrast to wider and/or more tortuous arteries. The same inference was supported by Taddei, 6 who stated that a distinction should be made between macro-circulation (large arteries) and microcirculation (narrower arteries). These two vessel types are subject to different types of regulation, so results obtained with large arteries cannot be extrapolated to the microcirculation. This agrees with our hemodynamic model and its histological verification in the present study. 

 Biomechanical forces such as low shear stress due to disturbed blood flow activate the endothelium and thus increase vasomotor dysfunction and promote inflammation by upregulating pro-atherogenic genes, 3 causing atherosclerosis. Plaques are predominately formed at specific sites within the arterial tree, suggesting a critical role for local factors within the vasculature. Atherosclerotic plaques are found at arterial bifurcations and branch points 1 and in wide blood vessels. It was recognized by Caro et al. 13 that sites of lesion development responsible for atherosclerosis were associated with regions of altered blood flow. This was also established by the microanatomical study appended below. 

### Microanatomical analysis

 We now discuss the development of atherosclerosis, as interpreted from the slides taken from sites of anatomical anomalies, namely, at bends/siphons, branching points in ECA/ICA and in the wider CCA, on the basis of our hemodynamic model developed in the material and methods section. The plaque layers of varying thickness, ranging from 7 to 79 mm at CCA-1, CCA-2 in the straight part and CCA-3 at the bifurcation ( Figure 1 ) with larger area of cross section (314 mm^2^) were deposited because the computed drag stress was lower, 0.44 F_d_ dyne/cm^2^ (from equation-1). The thickness of atherosclerosis was between 1.0 and 2.5 mm in the ECA at the first bend ( Figure 2 A), associated with a bifurcation with greater shear stress computed at 1.20F_d_ . The lower thickness of plaque could be due to sudden reduction in radius/area of the ECA from the CCA. In ECA-2, at the point of separation of the lingual artery from ECA, the thickness of atherosclerosis increased in the range 2-5 mm ( Figure 2 B), probably because of the division of stress at the bifurcation. In ECA-3, at the third bend, the thickness of deposition of atherosclerosis increased, ranging from 1.5 to 8.0 mm ( Figure 2 C), owing to a drastic cumulative fall in stress, 0.72F_d_ units from 1.56F _d_. In ECA-4, at the point of origin of the occipital artery, the thickness was reduced to 1.0-3.0 mm ( Figure 2 D) owing to thin artery separation and reduction in the diameter of the artery causing increased stress and velocity. In ECA-5, at the fourth bend, atherosclerosis thickness increased to 2.0-11mm ( Figure 2 E) owing to an appreciable reduction in stress from 7.7 F_d_ (in the straight part) to 1.37F_d_. At ECA-6, in a straight part between the fourth and fifth bends, there was no development of atherosclerosis ( Figure 2 F) as the stress remained constant and consistent at 1.37F_d_. 

 In ECA-7, at the fifth bend, the atherosclerosis thickness was again between 2.0 and 10.0 mm ( Figure 2 G) as the stress fell to 1.2F_d_ from 1.37F_d_ because of this bend. This supports our model. In ECA-8, at the bifurcation, the thickness was in the range 2.0-8.0 mm ( Figure 2 H), and at ECA-9, at the bifurcation of the maxillary artery, the atherosclerosis thickness was from 1.5 to 2.5 mm ( Figure 2 I). This is attributable to the reduction in radius of the arteries. When the radius is reduced the stress will rise, but the division of blood flow and directional change at the bifurcation could cause a small amount of atherosclerosis. 

 In ICA-1, plaque thickness was 2.0-7.0 mm ( Figure 3 A) at an effective shear stress of 0.93F_d_ at the first bend. At the bifurcation of the CCA, the atherosclerosis thickness was between 7 and 15 mm in the ICA portion of the lumen at a shear stress of 0.32 F_d_. The reduction in plaque thickness could be due to an increase in effective stress to 0.93 F_d_. In ICA-2, the thickness increased to 2-15 mm ( Figure 3 B) owing to the fall in effective shear stress from 0.93 F_d_ to 0.62F_d_ due to the sharper/more tortuous second bend. Further, in ICA-3, the thickness increased to 22 mm ( Figure 3 C) with a continued shear stress of 0.62F_d_ in the straight part beyond the second bend. Detailed physical and histological slide observations are recorded in Table 1 . 

 This hemodynamic model theoretically bridges the relationship of atherosclerosis under variant hemodynamic conditions in the presence of anatomical anomalies as variations in the ECA and ICA. Our study, as elaborated above, was an attempt to establish this model by an experimental study of arterial microanatomy. The histological slides clearly revealed development of atherosclerosis at anatomical variations in shape, size, and course pattern, forming at bends, bifurcations and wide apertures of the ECA, ICA and/or CCA. The presence of atherosclerosis and hemodynamic changes due to the modified morphology of the ECA, ICA and/or CCA were consistent with the predictions. 

### Clinical significance

 The large aperture of the CCA and anatomical variants of ECA and ICA, forming bends and bifurcations, modify the functional anatomy/circulatory dynamics of blood flow according to our hemodynamic model. This in turn triggers accumulation of atherosclerotic plaque at these sites. Buildup of atherosclerotic plaques within the CCA, ECA and ICA is the underlying cause of most forms of strokes. Surgical interventions can create further complications in the presence of these anomalies if the surgeon is unaware of variations such as tortuosity. The pathological implication of tortuosity in the ICA/ECA necessitates not only microscopic data, 14 to be used for diagnosis and treatment of diseases related to tortuosity/bifurcations, but also a sound hemodynamic model to establish predisposition to atherosclerosis. 

 Atherosclerosis causing narrowing of the ECA/ICA can lead to hypertension. Small pieces of plaque can separate, causing thromboembolic symptoms and resulting in ischemia in structures supplied by the ECA /ICA. Turbulence in flow at bends/bifurcations can damage the internal fibers of the ECA/ICA causing dissecting hematoma. Carotid kinking can either be a source of cerebral emboli or the vessel may be temporarily occluded by head/neck rotation, causing symptoms of cerebral ischemia. 15 These changes may be caused by hemodynamic changes and/or thromboembolic mechanisms, flow changes due to mechanical occlusion resulting from changes in head position, microembolization, and flow stasis at the level of the kink. 16 ^,^ 17

 Surgical interest in the tortuosity of the ICA stems from the risk of exsanguinating hemorrhage during tumor resections and other procedures such as tonsillectomy, adenoidectomy, or oropharyngoplasty, particularly related to bleeding from the rich arterial supply to this region of the pharynx, including the ascending pharyngeal, lingual, facial, and maxillary branches of the ECA. 18

 A bend or loop in the ICA, described as an aberrant ICA, can be mistaken for a tumor or abscess and may cause injuries during routine ENT procedures such as tonsillectomy and drainage of a peritonsillar abscess. 19 ^,^ 20 Similarly, the bends in the ECA/ICA observed in the present study could also be interpreted as tumors or abscesses, creating complications during routine ENT procedures. 

Thus this study concludes that:

1. Velocity and dragging stress diminish at bends/siphons in tortuous arteries, branching points (ECA/ICA) and wider arteries (CCA) as a result of theoretical model and observational facts.
2. Development of atherosclerosis at bends/siphons in tortuous arteries, branching points (ECA/ICA) and wider arteries (CCA) was observed by physical examination and histological study.
3. This study is a highly useful investigation of the most dreaded disease, atherosclerosis, that causes cardiac arrest and strokes. The study is also essential for vascular surgeons during carotid endarterectomy or carotid-subclavian bypass surgeries. The authors strongly recommend extension of the study to coronary atherosclerosis and its relation with cervical siphon and branching points in large and medium-sized carotid arteries.

### Limitations

1. The photographs of slides were taken at varying zoom factors for better clarity, so the values of atherosclerotic thicknesses provide a representative trend of values rather than absolute values of thickness. In many cases, only part of the lumen could be photographed so complete thickness could not be measured.
2. The approximations in derivations of the formulae in the hemodynamic model will also provide qualitative interpretation rather than precise calculations.
3. More such experiments will reveal more insights into the subject. This single study may provide the search light for future investigations.
